# Multi-Criteria Evaluation for Sorting Motion Planner Alternatives

**DOI:** 10.3390/s22145177

**Published:** 2022-07-11

**Authors:** Georgios Papaioannou, Zaw Htike, Chenhui Lin, Efstathios Siampis, Stefano Longo, Efstathios Velenis

**Affiliations:** 1 Department of Engineering Mechanics, KTH Royal Institute of Technology, Teknikringen 8, SE-100 44 Stockholm, Sweden; 2 Advanced Vehicle Engineering Centre, School of Aerospace, Transport and Manufacturing, Cranfield University, Cranfield MK43 0AL, UK

**Keywords:** automated vehicles, motion planning, sorting alternatives, motion sickness, safety, energy efficiency, journey time

## Abstract

Automated vehicles are expected to push towards the evolution of the mobility environment in the near future by increasing vehicle stability and decreasing commute time and vehicle fuel consumption. One of the main limitations they face is motion sickness (MS), which can put their wide impact at risk, as well as their acceptance by the public. In this direction, this paper presents the application of motion planning in order to minimise motion sickness in automated vehicles. Thus, an optimal control problem is formulated through which we seek the optimum velocity profile for a predefined road path for multiple fixed journey time (JT) solutions. In this way, a Pareto Front will be generated for the conflicting objectives of MS and JT. Despite the importance of optimising both of these, the optimum velocity profile should be selected after taking into consideration additional objectives. Therefore, as the optimal control is focused on the MS minimisation, a sorting algorithm is applied to seek the optimum solution among the pareto alternatives of the fixed time solutions. The aim is that this solution will correspond to the best velocity profile that also ensures the optimum compromise between motion comfort, safety and driving behaviour, energy efficiency, journey time and riding confidence.

## 1. Introduction

Automated driving is considered one of the major technological developments within the automotive industry and is able to influence future mobility and improve life quality. Based on research surveys, there are suggestions that automated vehicles (AV) will constitute around 35% of vehicle sales, and 50% of all vehicle travel [1]. At the same time, there are important challenges, which could lead to the disuse of AV technology.

The ability to engage in other activities during the ride and the ability to use the commute time more productively is considered by consumers as one of the key reasons for the adoption of AVs [2,3]. However, all the envisaged AV designs, i.e., the handing over of vehicle control, seating backwards, or not having a clear view of the road ahead by displays or structures, provoke the incidence of motion sickness (MS) to the occupants [4]. Hence, a refocus on motion comfort is crucial when considering what is at stake.

Carsickness is motion sickness that results from provocative motion frequencies occurring in a road vehicle in transit, while vertical vibrations were considered one of the most important factors [5,6]. However, with the advent of AVs, the interest has been shifted towards the horizontal accelerations which occur during accelerating, braking and unexpected or intense directional changes. The reason is that the horizontal movement of AVs can be influenced by motion planning, allowing the engineers to design controllers which could enhance occupants’ comfort and mitigate motion sickness, as highlighted by Elbanhawi et al. [7].

In recent years, many different motion planning approaches have been developed due to the increased interest in AVs. Existing works can be broadly classified into three categories: simple geometric-based methods, heuristic-based methods and methods based on optimal control techniques [8]. Geometric-based and heuristic-based methods focus mainly on the generation of the path, while optimal control-oriented methods focus on the conversion of the computed path in a feasible trajectory by assigning a velocity profile to the path. The optimal trajectory is identified by the motion planning layer, which is responsible for computing a dynamically feasible trajectory according to the state model and the constraints. The cost function in these methods is selected based on the outputs of a vehicle model and considering the presence of obstacles that exist or not along the path.

Nowadays, the need to consider cost-functions related to the minimisation of motion sickness ( MS ) in trajectory planning studies has emerged. This is because all the envisaged designs of driverless vehicles will be completely different from the human driven vehicles (HDVs) and in contradiction with the occupant’s habituated driving experiences. Moreover, the users might perceive the AV’s driving style as more aggressive, as it might result in excessive, unexpected head and body motion. On the other hand, the excessive reduction in the speed as a measure to mitigate MS can negatively affect traffic [9], but most importantly comfort and acceptance, while the user’s dissatisfaction might also increase due to the longer travel times [10,11]. In recent literature, the focus has been the minimum time solutions either for lap time simulation cases [12] or minimum cornering of passenger vehicles [13]. Few works have considered the motion sickness metrics [14,15,16] as the main objective in motion planning, but more work has to be conducted. This is because it is crucial to obtain a trajectory that satisfies the model and the constraints, while it guarantees an optimal compromise between motion comfort and journey time.

Despite the importance of mitigating motion sickness without neglecting journey time, the optimum velocity profile with which the vehicle will finally drive should be selected after taking into consideration additional objectives. Firstly, the driving style should be smooth enough and not assertive during the ride, as high acceleration values and jerk will make the passenger feel discomfort [17] and the vehicle might be perceived as unstable, while it is not. Few researchers [18,19] used aggressive driving metrics as the main objective for the motion planning of an AV, while the passengers’ confidence in riding or subjective feel (i.e., how the vehicle is perceived to drive) has not been used. This could potentially affect the trust of the passengers towards the driving experience. Additionally, AVs are expected to have a significant impact on the decrease in fuel emissions, therefore, the energy efficiency should also be included in the final selection of the optimum velocity profile. As a result, various researchers [20] have used energy efficiency as their main objective in the motion planning studies, while Han et al. [21] investigated the fundamentals of energy efficient driving by formulating control problems. Last but not least, apart from constraints that secure the vehicle stability of the vehicle, it is important to consider it as an additional objective, as well by using appropriate metrics. To the authors’ knowledge, very few works have tried to combine many objectives in the past [22]. However, in these cases, the objectives were combined in the main cost function with weighting coefficients.

In this direction, this paper considers in a simplified scenario the employment of sorting algorithms to sort the alternatives provided by the motion planner after considering additional objectives. More specifically, this work presents the application of optimal control to extract the optimum trajectory to be considered as a reference from AVs. The problem, which is formulated, seeks the optimal velocity profile for a predefined road path for minimising the motion sickness ( MS ) at multiple fixed journey time ( JT ) solutions. As the optimal control is focused on the MS minimisation, a sorting algorithm is applied to seek the optimum solution among the pareto alternatives by considering the additional objectives. The aim is that this solution will correspond to the best velocity profile that ensures the optimum compromise among the motion comfort, the driving behaviour, the energy efficiency, the vehicle stability, the occupant’s confidence to ride and the journey time.

## 2. Background

### 2.1. Vehicle Model and Road Tracking

**Figure 1:**
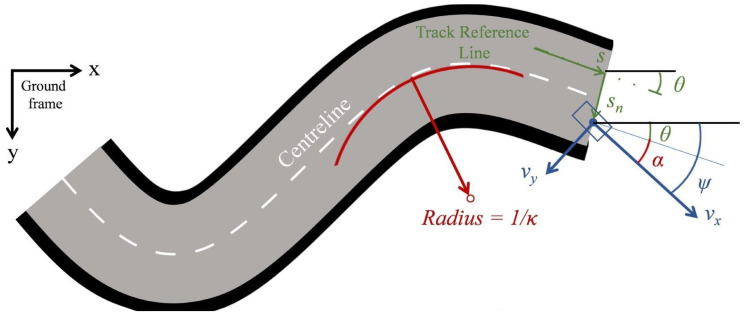
Curvilinear coordinates for road tracking.

In motion planning studies which use optimal control methods, one of the most commonly used and computationally efficient models is the point mass. The point mass model (Figure 1) is a simplified but robust vehicle model with kinematic equations of motion, which are as follows:

(1) $$\begin{array}{ccc} \ddot{x}=a_{x}, & \; & \ddot{y}=a_{y} \end{array}$$

where the inputs of this model are the longitudinal ( $a_{x}$ ) and lateral ( $a_{y}$ ) acceleration. Regarding the road tracking (Figure 1), the road path is considered similar to strips described by the *x* and *y* coordinates of the road centreline and lateral width ( $L_{w}$ and $R_{w}$ ). The road heading angle ( θ ), as well as x,y coordinates may be calculated by integrating the curvature as follows:

(2) $$\begin{array}{ccccc} \frac{d\theta }{ds}=\kappa \left(s\right), & \; & \frac{dx}{ds}=cos\theta , & \; & \frac{dy}{ds}=sin\theta \end{array}$$

The curvilinear coordinates approach has been proposed by Lot et al. [23], and is the most effective way to describe the road centreline using only the line curvature κ as a function of arc length *s* (Figure 1). The main advantage of the curvilinear coordinates approach is their use in tracking the orientation of the vehicle based on the calculus of the vehicle forward ( $v_{x}$ ) and lateral velocity ( $v_{y}$ ) according to Equations (3)–(5):

(3) $$\dot{s}=\frac{v_{x}cos\alpha -v_{y}sin\alpha }{1-s_{n}\kappa }$$

(4) $${\dot{s}}_{n}=v_{x}sin\alpha +v_{y}cos\alpha$$

(5) $$\dot{\alpha }=\dot{\psi }-\dot{s}\kappa$$

where $\dot{\psi }$ is the yaw rate; $s_{n}$ is the lateral offset on the road strip; and α is the vehicle relative heading to the road.

### 2.2. Road Paths and Profiles

**Figure 2:**
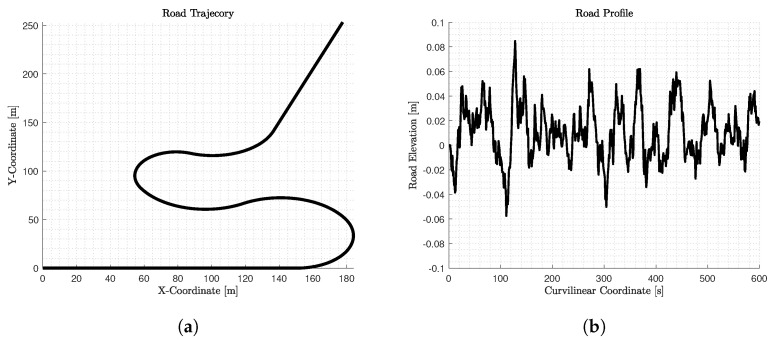
(**a**) The road path with the above X-Y trajectory and; (**b**) the Class B Road profile [24] assigned to it.

In this work, initially, we design a road path (Figure 2a) to seek the optimum velocity profile and then, a random road profile of Class B [24] (Figure 2b) is used to study in depth the vehicle dynamic behaviour using IPG/CarMaker 8.0.

## 3. Performance Metrics

Despite the importance of mitigating MS and minimising JT, the optimum velocity profile with which the vehicle will finally drive should be selected after taking into consideration additional objectives. Hence, in this work, we will also consider comfort, driving behaviour, energy efficiency, vehicle stability and subjective feel oriented objectives.

### 3.1. Motion Comfort-Oriented Metrics

Current tools for the assessment of passenger comfort and motion sickness include standardised metrics and models based on the direction, amplitude, frequency and duration of the accelerations experienced by the passenger. In this work, the illness rating of the passengers will be used to represent motion sickness.

#### 3.1.1. ISO-2631: Whole Body Vibrations

ISO-2631:1998 provides a guideline for the measurement and evaluation of human exposure to whole-body mechanical vibration and repeated shock. According to the standard, the ride comfort is assessed by combining the root mean square (RMS) values of the weighted accelerations ( $RC_{W_{i}}$ ) measured at the vehicle’s centre of gravity. More specifically, for each acceleration, either directional ( $\ddot{x}$ , $\ddot{y}$ and $\ddot{z}$ ) and rotational ( $\ddot{\phi }$ , $\ddot{\theta }$ and $\ddot{r}$ ) the weighted RMS value can be evaluated as follows:

(6) $$RC_{W_{i}}={\left(\frac{1}{t}\int_{0}^{t}a_{W_{i}}^{2}d\tau \right)}^{\frac{1}{2}}$$

where *i* refers to the type of the acceleration, either translational ( $\ddot{x}$ , $\ddot{y}$ and $\ddot{z}$ ) or rotational (*i*= rx for $\ddot{\phi }$ , ry for $\ddot{\theta }$ and rz for $\ddot{r}$ ); $a_{W_{i}}$ stands for the weighted accelerations in the time domain. The weighting of the accelerations is conducted based on ISO-2631:1998 [25], and more specifically regarding comfort, *W* ${}_{k}$ and *W* ${}_{e}$ are used for the vertical and the rotational accelerations, respectively. The overall ride comfort metric is evaluated by summing all the $RC_{W_{i}}$ , after multiplying each by appropriate factors ( $k_{i}$ ) based on the following equation:

(7) $$RC={\left(\sum_{i=1}^{6}k_{i}^{2}RC_{W_{i}}^{2}\right)}^{1/2}$$

where $k_{i}$ is the multiplying factor for each term (*i* = *x*, *y*, *z*, rx , ry and rz ).

#### 3.1.2. Illness Rating

ISO-2631:1998 provides an empirical approximation for assessing motion sickness provoked from the vertical motion ( $MSDV_{z}$ ), which mainly is a simplification of Equation (7) and is derived by giving a non-zero value to $k_{z}$ only:

(8) $$MSDV_{z}=k_{z}\times RC_{Wz}$$

This metric ( $MSDV_{z}$ ) occurred from experiments related with the sea sickness from the vertical motion [26,27]. Later, Turner et al. [28,29] proved its suitability for road vehicles as well, while they validated it for approximating the motion sickness likelihood in the horizontal direction. The motion sickness that occurred from the lateral (*x*-axis) and longitudinal (*y*-axis) motion ( $MSDV_{x,y}$ ) could be evaluated from Equation (9), which is also a simplification of Equation (7) by giving a non-zero value only to $k_{x}$ and $k_{y}$ ( $k_{x}$ , $k_{y}$ ≠ 0 and $k_{rx}$ , $k_{ry}$ , $k_{rz}$ , $k_{z}$ = 0).

(9) $$MSDV_{xy}=k_{x}\times RC_{Wx}+k_{y}\times RC_{Wy}$$

For the calculation of *RC* ${}_{W_{x}}$ and *RC* ${}_{W_{y}}$ , the accelerations are weighted, but this for the illness rating based on ISO-2631:1998 but with the *W* ${}_{f}$ weighting filter. The $MSDV_{z}$ metric illustrated a linear regression with the mean passenger illness rating, leading to the following equation for the assessment of the passengers predicted illness rating ( IR ):

(10) $$IR=K\times MSDV_{z}$$

where *K* is an empirically derived constant (= 1/3 ) according to data obtained from motion sickness-related studies in seat and road transport. In this work, it will be assumed that this also stands for the horizontal accelerations. So, the IR metric will be calculated as follows, and will be considered as a comfort-oriented objective:

(11) $$IR=K\times MSDV_{xy}$$

### 3.2. Aggressive Driving

In principle, the driver’s aggressiveness should be measured by how fast the driver accelerates and decelerates. To evaluate the levels of aggressive driving ( AD ), the jerk of the longitudinal acceleration ( $\ddot{x}$ ) is normally used. The jerk ( $J_{a_{i}}$ , Equation (12)) is defined as the rate of change in acceleration and deceleration, having a significant impact on the safety and comfort of passengers [30]:

(12) $$J_{a_{i}}=\frac{da_{i}}{dt}$$

where *i* is *x* and *y* for longitudinal ( $\ddot{x}$ ) and lateral acceleration ( $\ddot{y}$ ), respectively. An acceleration profile shows how a driver speeds up and slows down, whereas a jerk profile shows how a driver accelerates and decelerates. The latter is more important in determining drivers’ aggressiveness. In this work, the sum of $J_{\ddot{x}}$ and $J_{\ddot{y}}$ RMS values (AD) are used as a metric of aggressive driving, according to Equation (13):

(13) $$AD={\left(\frac{1}{t}\int_{0}^{t}J_{\ddot{x}}^{2}d\tau \right)}^{\frac{1}{2}}+{\left(\frac{1}{t}\int_{0}^{t}J_{\ddot{y}}^{2}d\tau \right)}^{\frac{1}{2}}$$

### 3.3. Energy Efficiency-Oriented Metrics

One factor which has significant effect on vehicle fuel consumption is the rate at which the vehicle is accelerated, as studies have shown that rapid or frequent accelerations result in increased consumption. The total energy demanded from the vehicle over any cycle is the time integral of the power requirement:

(14) $$E=\int_{T_{i}}^{T_{f}}Pdt$$

where *E* is the total energy demand; *P* the instantaneous power requirement; $T_{i}$ the initial time and $T_{f}$ the final time. The power required could be expressed as the product of the instantaneous force produced by the propulsion motor ( $F_{m}$ ) and the velocity of the vehicle (*v*):

(15) $$E=\int_{T_{i}}^{T_{f}}F_{m}\times v\left(t\right)dt=\int_{T_{i}}^{T_{f}}\left(m\times \frac{dv}{dt}+F_{r}\right)v\;dt$$

In the above equation, the force produced by the propulsion motor $\left(F_{m}\right)$ consists of two terms, the first $\left(m\frac{dv}{dt}\right)$ represents the inertial effect and the second ( $F_{r}$ ) denotes the resistive force (i.e., aerodynamic drag and rolling resistance). More specifically, $F_{r}$ is defined:

(16) $$F_{r}=\frac{1}{2}s\rho_{a}c_{d}A_{f}v{\left(t\right)}^{2}+c_{r}mg$$

where $\rho_{a}$ is the air density; $c_{d}$ denotes the aerodynamic drag coefficient; $A_{f}$ the vehicle’s frontal area and $c_{r}$ the rolling resistance coefficient. In this work, the minimisation of the energy demand (Equation (16)) will be considered through the metric of energy efficiency ( EE ).

### 3.4. Vehicle Stability-Oriented Metrics

#### 3.4.1. Vehicle Handling

Suspension travel is an important metric that indicates vehicle handling, as it depicts the ability of the system to support the vehicle’s static weight. The vehicle is well supported if the rattle space requirements are kept small. So, the maximum value of the suspension travel is usually selected as an index to assess the vehicle handling based on Equation (17):

(17) $$ST_{i}=max\left(\mathrm{Suspension}\;\mathrm{Travel}\right)$$

where i=FR,FL,RRandRL refers to four vehicle suspension systems, i.e., front right ( FR ) and left ( FL ), rear right ( RR ) and left ( RL ), respectively. The detailed equations for the suspension travel for various vehicle models can be found in Papaioannou et al. [31]. In this work, the sum of the maximum suspension travels at the two wheels of the $j^{th}$ axle, as shown below, is used as a metric of vehicle handling:

(18) $$ST\left[J\right]=max\left(ST_{[J]R}\right)+max\left(ST_{[J]L}\right)$$

where *J* is the front (*F*) and rear (*R*) axle.

#### 3.4.2. Rollover Stability

The load transfer at each axle ( $LTR_{i}$ , with i=R,F ) is used in order to evaluate the dynamic roll stability of the vehicle, using Equation (19) [32]:

(19) $$LTR\left[i\right]=\frac{F_{{ztR}_{i}}-F_{{ztL}_{i}}}{F_{{ztR}_{i}}+F_{{ztL}_{i}}}$$

LTR is used to assess the rollover propensity of the vehicle by considering the vertical tyre forces $F_{ztR}$ and $F_{ztL}$ . This index ranges from −1 to 1 and identifies when either the right or the left wheel has lost contact with the ground. When LTR[i] is close to −1 or 1, then the right or the left wheel of the $i^{th}$ axle is close to experiencing lift off, respectively. In this work, the maximum of the absolute values will be used to access the rollover propensity, which will illustrate if either the front or the rear axle has lifted-off.

(20) MLTR[i]=max(|LTR[i]|);

### 3.5. Riding Confidence-Oriented Metrics

**Figure 3:**
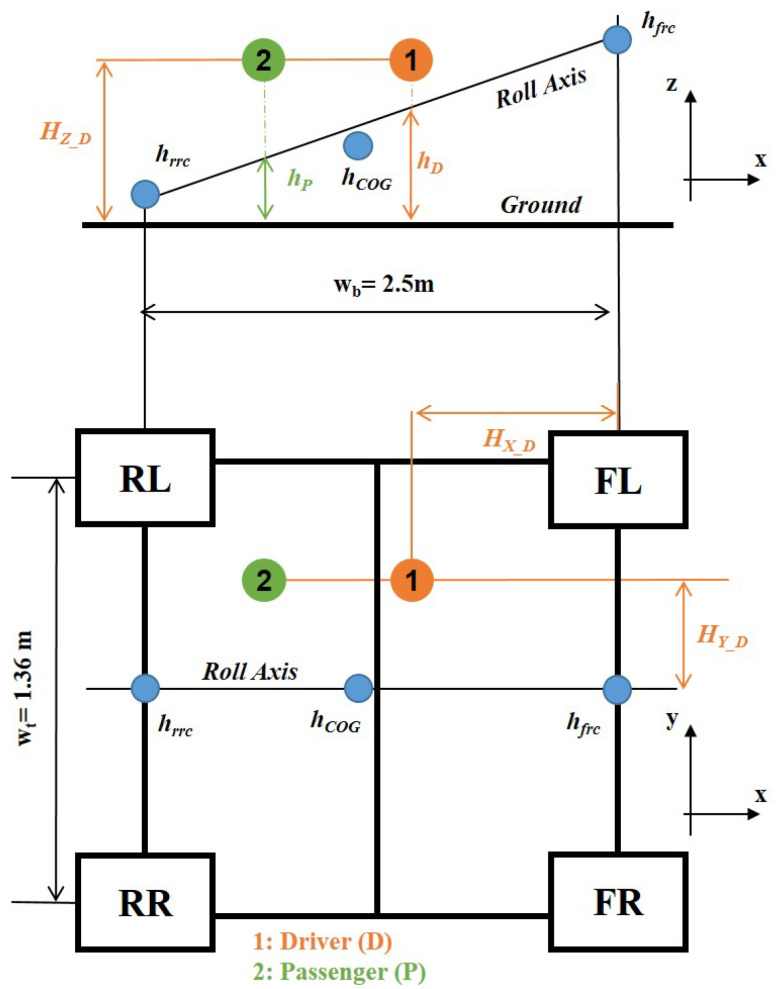
Vehicle side (top figure) and top (bottom figure) view.

Until now, vehicles have been driven by people and are considered machines to be felt by the driver. For the subjective evaluation of the driver-feel, three main parameters are considered and consist of the confidence drive level, the safe vehicle behaviour and the fun to drive [33]. In AVs, two of the three objectives could change. The confidence drive level could be neglected considering the lack of a driver, and the fun to drive could be transformed to “fun to ride”, considering the subjective feel of the passengers and how they perceive the ride in addition to the motion comfort. A metric able to capture the subjective feel of the occupants is the perceptible roll index ( $SF_{i}$ ), as proposed by Trivedi et al. [34]. The metric ( $SF_{i}$ ) combines the most common metric for roll performance, i.e., the roll gradient and the position of the passengers. The $SF_{i}$ metric is derived by Equation (21), and when this value is increased more motion is felt by the occupant:

(21) $$SF_{i}=\Phi \frac{\pi }{180}q$$

where *i* is equal to *D* or *P*, referring to the subjective feel perceived by the occupant in the driver’s or the passenger’s position ( H−point ), respectively, as shown in Figure 3; Φ is the roll gradient; and *q* is the rotational arm of the occupants H−point . The H−point is the position of the occupant’s hip measured from the front axle ( X−axis ), the centre plane of the vehicle ( Y−axis ) and the road ( Z−axis ). Regarding the rotational arm (*q*), it is derived from Equation (22):

(22) $$q_{i}=\sqrt{H_{y_{i}}^{2}+{\left(H_{z_{i}}-h_{i}\right)}^{2}}$$

where $H_{y_{i}}$ and $H_{z_{i}}$ are the driver’s ( i=1 ) and the passenger’s ( i=2 ) H−point coordinates in the *Y* and the *Z* axis, respectively. The height of roll axis ( $h_{i}$ ) at $H_{x_{i}}$ distance from the front axle ( H−point plane ) is defined as follows:

(23) $$h_{i}=hrrc+\frac{hfrc-hrrc}{wb}H_{x_{i}}$$

where hfrc and hrrc are the roll center heights at the front and rear axle.

## 4. System Design

The goal is to find the appropriate vehicle control inputs, that can drive the vehicle along a predefined path from the initial position ( $s_{0}$ ) to the final position ( $s_{f}$ ), such that the motion sickness, represented by illness rating ( IR ), to be minimised for various fixed journey time ( JT ) cases. This problem could be codified as an optimisation problem with the optimal trajectory and velocity profile of the vehicle to be its solution. GPOPS software [35] will be used for this. Afterwards, a sorting algorithm ( k−ε ) will be used to seek the optimum solution among the alternatives by considering additional objectives. This approach of selecting a main objective for the main optimisation procedure (i.e., the OCP) and adding additional objectives in the sorting algorithms have been used successfully by Papaioannou et al. [36,37] in the optimisation of passive and semi-active vehicle suspensions.

### 4.1. Optimal Control Problem (OCP)

#### 4.1.1. Dynamic Model, States and Control Inputs

The state space form of our dynamic system, i.e., vehicle model (MDL), can be written as follows:

(24) $$\dot{x}{}_{1}=f_{{MDL}_{t}}\left[x_{1}\left(t\right),u_{1}\left(t\right)\right]$$

(25) $$u_{1}={\left[a_{x}\left(t\right),a_{y}\left(t\right)\right]}^{T}$$

(26) $$x_{1}={\left[v_{x}\left(t\right),v_{y}\left(t\right),s\left(t\right),s_{n}\left(t\right),\alpha \left(t\right),x\left(t\right),y\left(t\right),\theta \left(t\right)\right]}^{T}$$

where $f_{{MDL}_{t}}$ is the function representing the equations of motion in the time domain; $u_{1}$ are the two control inputs (Equation (25)); $x_{1}$ are the eight state variables (Equation (26)).

The independent variable of the problem, i.e., the elapsed time ( $t_{f}$ ), should be changed to the distance (*s*) in order for us to be able to add the elapsed time in the formulation of the problem either as cost function (journey time- JT ) or as a state variable for fixed time scenarios. Considering that in this work we will investigate the second case, the general state space form of the dynamic system (Equation (24)), will be transformed in the distance domain (Equation (27)) using the derivation rule (Equation (28)) for each state:

(27) $$x_{2}^{\prime }\left(s\right)=f_{{MDL}_{s}}\left[x_{2}\left(s\right),u_{2}\left(s\right)\right]$$

(28) $$\frac{d\zeta }{ds}=\zeta^{\prime }=\frac{d\zeta }{dt}\frac{dt}{ds}=\dot{\zeta }{\dot{s}}^{-1}$$

where $f_{{MDL}_{s}}$ is the function representing the equations of motion in the distance domain; $x_{2}$ (s) and $u_{2}$ (s) are the states and the control inputs in the distance domain, respectively; ζ is any state of $x_{2}$ . In the s domain, the inputs are the same and only the time (*t*) is included in the states of our optimal control problem to study fixed time solutions. The states are illustrated below:

(29) $$x_{2}={\left[v_{x}\left(s\right),v_{y}\left(s\right),s_{n}\left(s\right),\alpha \left(s\right),x\left(s\right),y\left(s\right),\theta \left(s\right),t\left(s\right)\right]}^{T}$$

#### 4.1.2. Cost Function

The OCP seeks the appropriate control inputs to minimise the cost function ( $J_{c}$ ) that describes the objective of the problem, i.e., motion sickness, as presented in Equation (30):

(30) $$J_{c}=\Lambda \left[x_{2}\left(s_{0}\right),x_{2}\left(s_{f}\right)\right]+\int_{s_{0}}^{s_{f}}L\left[x_{2}\left(s\right),u_{2}\left(s\right)\right]$$

where Λ consists of terminal costs that are not considered in this work, while the second term will represent motion sickness by incorporating the $MSDV_{xy}$ , as illustrated in Equation (9), in the definition of the cost function. Therefore, Equation (30) will be transformed into Equation (31):

(31) $$J_{c}=k_{x}\int_{s_{0}}^{s_{f}}a_{x}^{2}\left(s\right)ds+k_{y}\int_{s_{0}}^{s_{f}}a_{y}^{2}\left(s\right)ds$$

#### 4.1.3. Constraints

Considering the above, the motion planning problem has formulated as shown in Equations (32)–(35):

(32) $$u^{*}\left(\cdot \right)=\underset{u(\cdot )}{arg min}J_{c}\left(x_{2}\left(s\right),u_{2}\left(s\right)\right)$$

(33) $$subject\;to:x_{2}^{\prime }\left(s\right)=f_{MDL_{s}}\left(x_{2},u_{2}\right)$$

(34) $$\sigma (x_{2},u_{2},s)⩽0$$

(35) $$b(x_{2}\left(s_{0}\right),x_{2}\left(s_{f}\right))=0$$

where σ and *b* are inequality and equality constraints that can configure the scenario of the optimal control problem. More specifically, in this work, in order to secure the fixed journey time ( $T_{demand}$ ), the final time ( $t_{f}$ ) is set to be equal to $T_{demand}$ (Equation (36)). Then, two inequality constraints, as described in Equation (37), are considered.

(36) $$t_{f}=T_{demand}$$

(37) $$\sqrt{{\ddot{x}}^{2}+{\ddot{y}}^{2}}⩽a_{max},-L_{w}⩽s_{n}⩽R_{w}$$

The first inequality constraint (Equation (37)) ensures that the vehicle will be able to accelerate within the bounds ( $a_{max}$ ) set by the friction circle [38]. This constraint secures the vehicle stability. Moreover, with the second inequality constraint, the vehicle model is bounded to never exceed the road borders considering left-width ( $L_{w}$ ) and right-width ( $R_{w}$ ) from the centreline of the road (Equation (37)). However, in this work, both will be zero ( $R_{w}$ = $L_{w}$ = 0), forcing the vehicle to follow the centreline of the road. Last but not least, boundary conditions regarding the vehicle velocity have been added, in order to achieve the most feasible optimal solution, with the overall minimum and maximum velocity being set at $u_{min}$ = 0 [m/s] and $u_{max}$ = 30 [m/s], respectively.

### 4.2. Multi-Criteria Decision Making

#### 4.2.1. Pareto Front

The optimisation is described as a problem of minimisation of objective functions. In single objective optimisation problems, the focus is turned on a scalar number, while in multi–objective optimisation (MOO) the objective function is a vector and there is not a single solution that optimises the problem. When the objective functions are in conflict in MOO problems, an infinite number of solutions exists shaping the Pareto front, which finally presents the trade-offs in compromising the different objectives. In this work, in order to generate a Pareto set of optimal solutions, the formulated single objective optimal control problem for minimising MS will be solved for different fixed time solutions ( $T_{{demand}_{i}}$ ).

#### 4.2.2. Sorting Algorithm *k* − ε

The solutions of the Pareto set are equally good and satisfy different subjective preferences, while the number increases as the complexity of the problem formulation is increased. In this work, we will apply the k−ε sorting algorithm [39], which is able not only to vet solutions taking into consideration if an objective is or not better by another, but also to quantify the entity of this variation. Through the k−ε optimality method, solutions which “have something more” than the others are identified and proposed to the designer. More specifically, according to this method, all the Pareto solutions are k− optimal. Thus, if k=0 stands for a solution, then it is just Pareto optimal, whereas if k=n−1 , where *n* is the objectives number, then the so called “utopia point” is identified and is the global optimum. The *k* levels are evaluated according to Equation (38).

(38) $$k=min_{Z}\left(\sum_{i=1}^{n}\Gamma \left(\Delta f_{i}\right)\right)-1$$

where $\Delta f_{i}$ is the difference between the $i^{th}$ objective of the considered solution compared to a different Pareto optimal solution; Γ(x) is a merit function evaluated based on Equation (39). In order k−ε to seek the "something more" than the others, an indifference threshold ε is included in the merit function. So, if the difference $\Delta f_{i}$ is lower than ε , selected by the designer, the solution is not sorted out as in other methods. The use of this threshold offers a continuous degree of optimality in the solutions.

(39) $$\begin{array}{c} \Gamma \left(\Delta f_{i}\right)=\left\{\begin{array}{cc} 0, & \Delta f_{i}\geq \varepsilon \\ 1-\frac{\Delta f_{i}}{\varepsilon }, & 0<\Delta f_{i}<\varepsilon \\ 1, & \Delta f_{i}\leq 0 \end{array}\right. \end{array}$$

## 5. Results and Discussion

**Figure 4:**
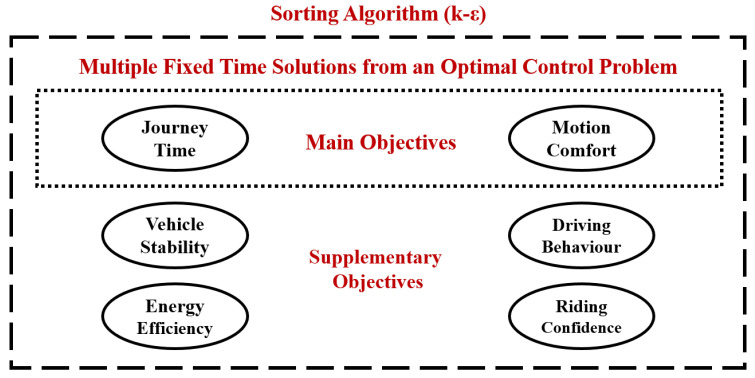
The combination of OCP and *k* − ϵ algorithm to identify the optimum velocity profile among multiple alternatives.

In this work, two optimisation algorithms are combined to seek the optimum velocity profile among the alternatives that have shaped a Pareto front and consist of optimal solutions of OCP problems with different fixed time for the minimisation of motion sickness. More specifically, the procedure is described by Figure 4 and is divided in three steps.

**Figure 5:**
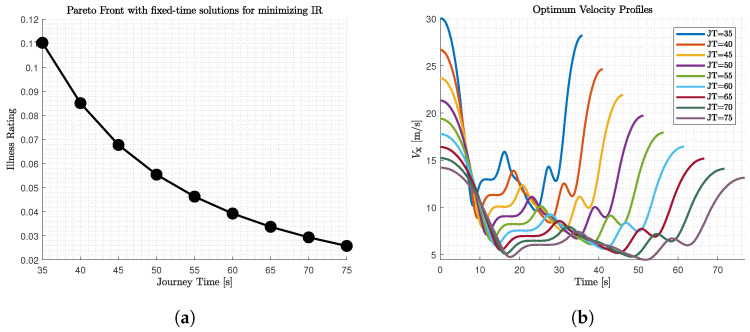
(**a**) The Pareto front with the optimal solutions obtained from the OCP for different fixed time cases ( $T_{{demand}_{i}}$ ) for the minimisation of IR and (**b**) their corresponding optimal velocity profiles.

**Figure 6:**
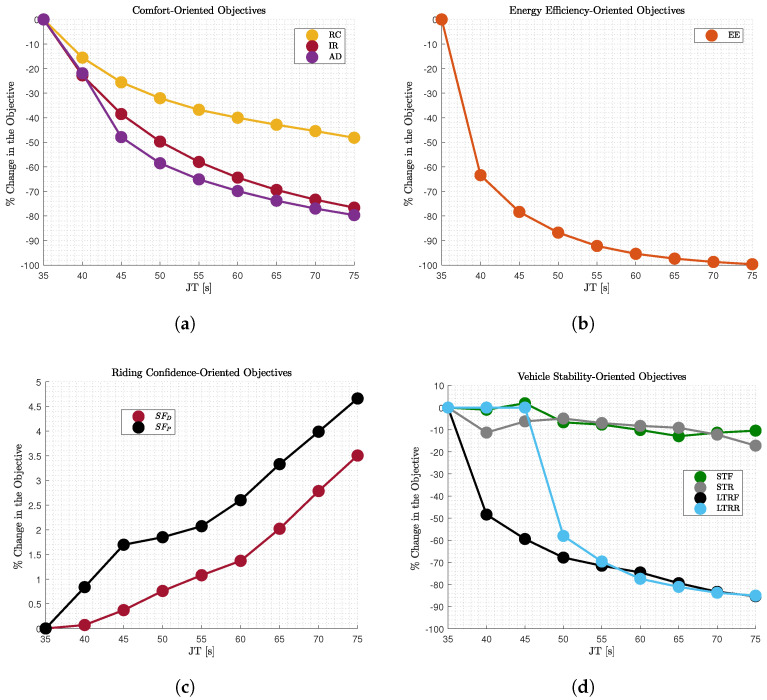
Additional (**a**) comfort-oriented, (**b**) energy efficiency-oriented, (**c**) riding confidence, (**d**) vehicle stability-oriented performance metrics evaluated by the outputs of an IGP/CarMaker 8.0 vehicle model following the predefined path with the assigned velocity obtained by the OCP (Figure 5b) for different fixed time cases.

- Firstly, an optimal control problem is formulated for multiple fixed journey time (JT) solutions and is solved using GPOPS II solver with MATLAB suite [35]. After obtaining all the optimal solutions, a Pareto Front (Figure 5a) is generated.
- Secondly, after having obtained the velocity profiles (Figure 5b) for various fixed solutions, a commercial software (IPG/CarMaker 8.0) is used to follow the predefined path with the assigned velocity in order to evaluate more performance aspects of the vehicle behaviour and the passengers condition (Figure 6). In order to achieve it, the lateral control for path-following is realised taking advantage of IPG Driver, which is a closed-loop control algorithm provided by the software. Moreover, a PID controller is utilised for the longitudinal control and the velocity tracking. According to Figure 6, the vehicle stability is considered as an additional objective, but the vehicle stability is already secured through constraints in the OCP. The supplementary objective is added to further secure it with additional metrics that refer to ride dynamics and rollover propensity.
- Finally, the k- ε algorithm, a sorting algorithm for multi-decision criteria making, is applied to seek the optimum solution among the Pareto alternatives considering the additional objectives. Prior to this, in order to generate more alternatives of JT , the Pareto Fronts of all the objectives with regards $JT=T_{{demand}_{i}}$ are interpolated for $T_{{demand}_{i}}^{\prime }$ .

### 5.1. Optimal Control Problem

Regarding the first part of this work, the minimisation of motion sickness is investigated for a set of fixed journey time cases (i.e., $JT=T_{{demand}_{i}}$ ), as shown in Equation (40).

(40) $$JT=T_{{demand}_{i}}\in \left[35,75\right]$$

with an interval of 5 s. The road path selected is fixed without allowing any lateral manoeuvrability to the vehicle by setting the road width at zero (i.e., the road boundary of left border and right border measured from the centreline $L_{w},R_{w}=0$ m). The output of these solutions is plotted shaping the Pareto front (Figure 5a) and illustrating the conflicting relation of our objectives. According to Figure 5a, the IR metric decreases with higher rate in the first three cases (around 22% per 5 s increase until 50 s), while afterwards the decrease is less (around 16%). The above remark is also depicted in the optimal velocity profiles assigned in the path (Figure 5b) for each JT case. According to Figure 5b, all the optimal velocity profiles follow the same pattern, as they are assigned to the same path, but with harsher and more aggressive accelerations when the JT is smaller.

### 5.2. Additional Objectives

As described previously, the optimal velocity profiles are assigned to the predefined path with higher accuracy and assess more performance aspects, which have been described in Section 3. The fraction of change of each metric with regards to the corresponding value of the fastest case ( JT=35 s) is plotted versus journey time ( JT ) in Figure 6. The additional metrics are divided into four groups of metrics, where the one referring to (A) motion comfort ( RC and IR ) and driving behaviour ( AD ) is illustrated in Figure 6a, (B) to energy efficiency ( EE ) is illustrated in Figure 6b, (C) to vehicle stability ( STF , STR , LTRF and LTRR ) is illustrated in Figure 6d, and (D) to riding confidence ( $SF_{D}$ and $ST_{P}$ ) is illustrated in Figure 6c. The pattern illustrated in these figures presents the relation of each metric with JT , when assigning different velocity profiles to a predefined path, and this would be the shape of their Pareto Front with regards to JT , if their value was plotted instead of the fraction of decrease.

Regarding the comfort-oriented metrics (Figure 6a) a conflicting relation with the journey time is illustrated as expected. The increase in the journey time leads to smoother acceleration and deceleration and hence, the comfort perceived by the occupants is increased. Based on the comparison of the various metrics ( RC , IR and AD ), we can extract conclusions about the efficiency of our cost function ( IR ). According to Figure 6a, the decrease that occurred in AD is greater than IR , which served as our cost function in the OCP formulation. On the other hand, the RC metric illustrates a smaller decrease compared to the cost function for the various JT cases, as it considers additional terms such as the vertical accelerations, as shown in Equation (7). As far as the energy efficient metric (Figure 6b) is concerned, it illustrates a conflicting relation with JT as well. The increase in the JT from 35 s to 40 s offers a significant decrease of 65% in the vehicle’s energy consumption, while afterwards the decrease is much less for the larger JT cases, i.e., after 60 s we identify 2% for each interval. Regarding the riding confidence-oriented metrics are concerned (Figure 6d), they have a non-conflicting relation with the JT , so $SF_{D}$ and $SF_{P}$ evaluated in the driver and passenger position is increasing as the JT is increased. Finally, the vehicle stability-oriented metrics (Figure 6) have a more complicated relation with JT . More specifically, MLTRR and MLTRF illustrate a conflicting relation with JT , with the MLTRF to be constantly decreasing and offering a more stable front axle without the risk of lift-off as the JT is increasing. On the other hand, MLTRR does not improve in the first JT cases ( JT≤45 s), which means that the rear axle of the vehicle continues to experience lift-off in one of its wheels during the journey in these cases. However, after some point ( JT≥45 s) the rear axle of the vehicle is becoming more stable. Similar complicated relation with JT exists in STF and STR , which illustrate irregular variations but small (10%) while the JT is increased.

### 5.3. Sorting Algorithm k − ε

Having evaluated the additional objectives for the all the JT cases, the Pareto Fronts of all the additional objectives are interpolated for $T_{{demand}_{i}}^{\prime }$ , where $T_{{demand}_{i}}^{\prime }$ = [35:0.1:75], in order to generate more alternatives. Afterwards, the k- ε algorithm is applied to seek the optimum solution among the Pareto alternatives considering the additional objectives described in Section 5.2. More specifically, the objective function of k- ε is defined as follows:

(41) $$f=[RC,\;JT,\;AD,\;EE,\;STF,\;STR,\;ST_{D},\;SF_{P},\;MLTRF,\;MLTRR]$$

where RC is based on Equation (7); JT refers to the journey time of each optimal solution (Equation (40); AD is based on Equation (13); EE is the root mean square value of Equation (15); STF and STR are based on Equation (18) for the front and rear axle, respectively; MLTRF and MLTRR are based on Equation (19). The objective function is then used to calculate the *k* levels of each alternative based on Equation (38). Finally, the merit function from Equation (39) is calculated using the following threshold ( ε ):

(42) $$\varepsilon =P_{i}\left[max\left(f_{1}\right),...,max\left(f_{n}\right)\right]$$

**Figure 7:**
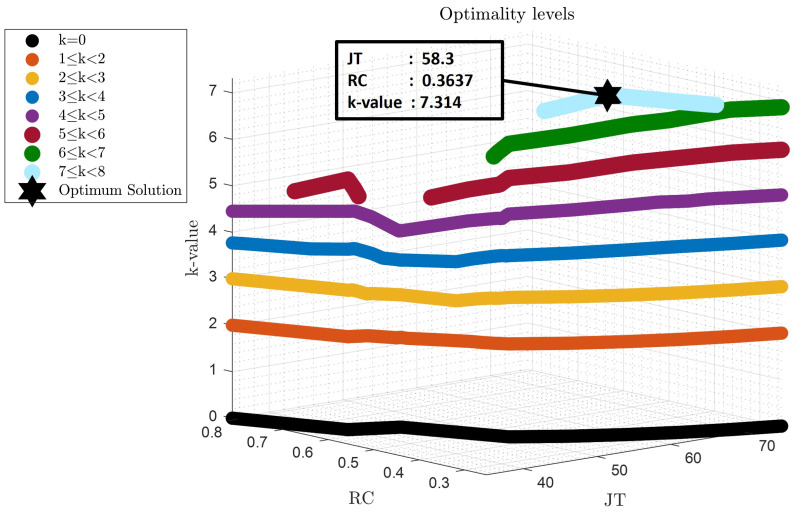
Optimum solution obtained by the sorting algorithm considering multiple design criteria ( RC , AD , EE , STF , STR , LTF , LTR , $SF_{D}$ and $SF_{P}$ ).

According to Figure 7, the optimum solution, which has managed to compromise all the objectives including in Equation (41), is located at JT = 58.3s . The *k*-value of this solution is 7.3 , which means it is dominating the rest of the objectives by 7.3 out of 9 (the value that the utopia point should have). More specifically, the solution converged close to the middle of the Pareto Frontiers which is the solution that compromises effectively all the conflicting relations that were illustrated in the previous section.

## 6. Conclusions

To sum up, in this paper, an OCP problem was formulated to seek the optimal velocity profile for minimising motion sickness at multiple fixed time solutions. The employment of IPG CarMaker proved the feasibility of the solutions and allowed investigating the impact of increasing the journey time to multiple performance aspects. The quantification of this impact outlined the importance of considering them as well in the motion planning process. Then, an approach combining two optimisation algorithms, i.e., the OCP and the k−ε method, is applied successfully to seek the best velocity profile that ensures the optimum compromise between motion comfort and driving behaviour, energy efficiency, vehicle stability, occupants confidence to ride and journey time. The application of these two algorithms aimed at two goals. The first goal was to investigate the sorting of the alternatives that the motion planner can provide, and secondly, to pave the path for investigating real-time applications of such methods. However, in this work, the employment of IPG CarMaker to evaluate the additional objectives, was selected due to the offline nature of the work. Such models are too computationally expensive and cannot be considered for real-time planning. Work is in progress to employ simplified models to evaluate the additional objectives, and employ this method in real-time planning. 

## Data Availability

Not applicable.
